# Supplementary material for: Empirical methods for controlling false positives and estimating confidence in ChIP-Seq peaks
Source: BMC Bioinformatics. 2008 Dec 5;9:523. doi: 10.1186/1471-2105-9-523 (PMC2628906; doi:10.1186/1471-2105-9-523)
Supplement: Additional File 1 — A variety of html documents from the USeq web site detailing the available applications, their best usage, output file type descriptions, command line menus, etc. [file 1471-2105-9-523-S1.zip › USeqUserGuides/JavaDoc.zip!/JavaDoc/bioroot/DBUtil.html]

DBUtil


|  |  |  |
| --- | --- | --- |
| **Overview** \| **Package** \| **Class** \| **Use** \| **Tree** \| **Deprecated** \| **Index** \| **Help** | |  |
| **PREV CLASS**   **NEXT CLASS** | **FRAMES**    **NO FRAMES**     **All Classes** |  |
| SUMMARY: NESTED \| FIELD \| CONSTR \| METHOD | DETAIL: FIELD \| CONSTR \| METHOD |  |


---


## bioroot Class DBUtil

```
java.lang.Object
  bioroot.DBUtil
```

---

``` public class DBUtil extends java.lang.Object ```

Utility class to hold database relevant methods and fields, some from Hall and Brown
The following is a collection of stuff for interacting with a SQL database
First open a connection by calling connectMySQL, then get results with
executeSQL, then close the connection with closeConnection.

---

**Constructor Summary**

`DBUtil()`

`DBUtil(java.lang.String database)`


| **Method Summary** | |
| --- | --- |
| `static java.lang.String` | `buildSqlStatement(java.lang.String reagentType, UserBean user, DBUtil bioRoot, boolean descending)`             Creates a sql statement given a UserBean and a boolean as to whether to add a DESC to the order by where appropriate. |
| `boolean` | `canAlter(UserBean userBean, java.lang.String tableName, int id)`             Checks whether the user can modify or delete an item. |
| `boolean` | `canView(UserBean user, java.lang.String tableName, int id)`             Checks whether the user can view an item without creating a bean, just an id |
| `java.lang.String[]` | `canView(UserBean user, java.lang.String tableName, java.lang.String[] ids)`             Checks whether the user can view multiple items based on id. |
| `boolean` | `checkIP(java.lang.String IP)`             Checks to see if a given IP has exceeded the number of allowable failed logins. |
| `void` | `closeConnection()`             Closes the Connection and subsequently the Statement and ResultSet objects |
| `boolean` | `connectMySQL(java.lang.String database)`             Method to connect to the MySQL lab\_collection database and create a statement object |
| `java.lang.String` | `convertOrganismIdToName(int id)`             Converts and Organism id to and Organism name. |
| `int` | `convertOrganismNameToId(java.lang.String name)`             Converts and Organism name to and OrganismId. |
| `java.sql.ResultSet` | `executeSQL(java.lang.String sqlQuery)` |
| `java.sql.ResultSet` | `executeSQLAdvance(java.lang.String sqlQuery)`             Execute and advance the ResultSet. |
| `boolean` | `executeSQLUpdate(java.lang.String sqlQuery)`             Fires SQL Update statement returning true if no problems were encountered. |
| `java.lang.String[]` | `fetchCellTypeConcats(int id, UserBean user)`             Fetches concatinate of name and notes from CellType |
| `java.lang.String[]` | `fetchColumn(java.lang.String statement)`             Returns a String[] of the items in a particular column. |
| `java.lang.String[]` | `fetchConcatMakeIdLast(java.lang.String sql, int numColumns, java.lang.String divider, int lengthCutOff)`             Returns a concatinate of the results where each String in the String[] represents a results table row where each column entry is separated by the divider String. |
| `java.lang.String[]` | `fetchGeneConcat(int id, UserBean user)`             Fetches concatinate of name and notes from Gene |
| `java.lang.String[]` | `fetchGeneNames(int labGroupId)`             Fetches geneNames given a labGroupId, assumes connection to BioRoot+Database. |
| `IdNameMatch[]` | `fetchIdNameMatchArray(java.lang.String sql)`             Fetches an IndexNameMatch Array. |
| `java.lang.String` | `fetchLabGroupName(int id)`             Fetches an labGroupName given an id, assumes connection to BioRoot+Database. |
| `java.lang.String[]` | `fetchLabGroups(java.lang.String[] labGroups)`             Returns a sorted list of lab groups. |
| `java.lang.String[]` | `fetchMarkerConcats(int id)`             Fetches concatinate of name and notes from Marker |
| `java.lang.String[]` | `fetchMarkerNames()`             Fetches markerNames given an labGroupId, assumes connection to BioRoot+Database. |
| `java.lang.String[]` | `fetchMultipleCells(java.lang.String sql)`             Returns a String[] of the results. |
| `java.lang.String[]` | `fetchOrganismConcat(int id)`             Fetches concatinate of name and notes from Organism |
| `int[]` | `fetchReagentCounts(UserBean user, java.lang.String tableName)`             Fetches the number of reagents in the users labgroup, and the number owned by the user. |
| `java.lang.String` | `fetchReagentNames(int labGroupId, java.lang.String reagentType)`             Fetches the sorted reagent names associated with a labGroup. |
| `java.lang.String[]` | `fetchRecentReagentConcats(int userId, java.lang.String reagentName)`             Fetches concatinate of name and notes from Plasmids |
| `java.lang.String[]` | `fetchResultConcat(java.lang.String sql, int numColumns, java.lang.String divider, int lengthCutOff)`             Returns a concatinate of the results where each String in the String[] represents a results table row where each column entry is separated by the divider String. |
| `int[]` | `fetchSingleColumn(java.lang.String sql)`             Returns a int[] of the items in the first column. |
| `java.lang.String[]` | `fetchSingleColumn(java.lang.String tableName, java.lang.String columnName)`             Returns a String[] of the items in a particular column. |
| `java.lang.String` | `fetchUserName(int id, boolean firstNameFirst)`             Returns firstName lastName or lastName, firstName given a user id number |
| `java.lang.String` | `getCell(java.lang.String sql)`             Returns the first cell from an sql statement. |
| `int` | `getCellTypeId(java.lang.String name, int labGroupId)`             Fetches an tissueId given a tissue name and labGroupId (they aren't unique except within a labGroup) |
| `java.sql.Connection` | `getConnection()` |
| `int` | `getGeneId(java.lang.String geneName, int labGroupId)`             Fetches an geneId given a geneName and labGroupId (they aren't unique except within a labGroup), assumes connection to BioRoot+Database. |
| `java.lang.String` | `getGeneName(int geneId)`             Fetches an geneName given an geneId, assumes connection to BioRoot+Database. |
| `int` | `getLastInsertId(java.lang.String table)`             Returns the last inserted row auto increment number. |
| `int` | `getOrganismId(java.lang.String organismName)`             Fetches an organismId given a organismName, assumes connection to BioRoot. |
| `java.lang.String` | `getOrganismName(int organismId)`             Fetches an organismName given an organismId, assumes connection to BioRoot. |
| `static java.lang.String` | `getResult(java.sql.ResultSet result)` |
| `int` | `getResultSetInt(java.lang.String columnName)`             Use to avoid a null return when attempting to fetch a ResultSet.getInt(). |
| `java.lang.String` | `getResultSetString(java.lang.String columnName)`             Use to avoid a null return when attempting to fetch a ResultSet.getString(). |
| `java.lang.String` | `getResultString()`             returns the first column values from a result, for testing |
| `int` | `getRowsEffected()` |
| `boolean` | `isConnectionClosed()`             checks if the connection is open |
| `boolean` | `isNameUnique(java.lang.String tableName, java.lang.String name)`             Looks for a particular name in a table with a Name column. |
| `boolean` | `isNameUnique(java.lang.String tableName, java.lang.String name, int labGroupId)`             Looks for a particular name in a table with a Name column and a particular lab group. |
| `java.lang.String` | `tableLastModified(java.lang.String tableName)` |
| `boolean` | `updateCommonAccessFields(ReagentBean reagentBean, java.lang.String tableName)`             Just updates fields that are accessible by all lab group memebers. |
| `boolean` | `updateCommonAccessFields(ReagentBean bean, UserBean userBean, javax.servlet.http.HttpServletRequest request)`             Just update the commonly accessible fields: lastUser, comments |
| `void` | `updateLoginFailure(java.lang.String IP)`             Increments the numberFailures for an ip in the Login table if it exists or creates a new record. |

**Methods inherited from class java.lang.Object**

`equals, getClass, hashCode, notify, notifyAll, toString, wait, wait, wait`

**Constructor Detail**

### DBUtil

```
public DBUtil()
```

---


### DBUtil

```
public DBUtil(java.lang.String database)
```


**Method Detail**

### connectMySQL

```
public boolean connectMySQL(java.lang.String database)
```

:   Method to connect to the MySQL lab\_collection database
    and create a statement object

---


### executeSQLUpdate

```
public boolean executeSQLUpdate(java.lang.String sqlQuery)
```

:   Fires SQL Update statement returning true if no problems were encountered.

---


### tableLastModified

```
public java.lang.String tableLastModified(java.lang.String tableName)
```

---


### isNameUnique

```
public boolean isNameUnique(java.lang.String tableName,
                            java.lang.String name)
```

:   Looks for a particular name in a table with a Name column. Throws
    error if no name column. Returns true if nothing is found. Not specific to a particular
    lab group.

---


### isNameUnique

```
public boolean isNameUnique(java.lang.String tableName,
                            java.lang.String name,
                            int labGroupId)
```

:   Looks for a particular name in a table with a Name column and a particular
    lab group. Throws
    error if no name column. Returns true if nothing is found.

---


### executeSQL

```
public java.sql.ResultSet executeSQL(java.lang.String sqlQuery)
```

---


### getResult

```
public static java.lang.String getResult(java.sql.ResultSet result)
```

---


### getConnection

```
public java.sql.Connection getConnection()
```

---


### canAlter

```
public boolean canAlter(UserBean userBean,
                        java.lang.String tableName,
                        int id)
```

:   Checks whether the user can modify or delete an item. They are the owner of the reagent,
    or a super user and the item is owned by the lab group.

---


### canView

```
public boolean canView(UserBean user,
                       java.lang.String tableName,
                       int id)
```

:   Checks whether the user can view an item without creating a bean, just an id

---


### canView

```
public java.lang.String[] canView(UserBean user,
                                  java.lang.String tableName,
                                  java.lang.String[] ids)
```

:   Checks whether the user can view multiple items based on id.
    Returns the ids that can be viewed by the user.

---


### fetchIdNameMatchArray

```
public IdNameMatch[] fetchIdNameMatchArray(java.lang.String sql)
```

:   Fetches an IndexNameMatch Array. Use with getting info about genes or organisms,
    or anywhere you want just the index and name and want to match these.

---


### getOrganismName

```
public java.lang.String getOrganismName(int organismId)
```

:   Fetches an organismName given an organismId, assumes connection to BioRoot.

---


### getOrganismId

```
public int getOrganismId(java.lang.String organismName)
```

:   Fetches an organismId given a organismName, assumes connection to BioRoot.

---


### getGeneName

```
public java.lang.String getGeneName(int geneId)
```

:   Fetches an geneName given an geneId, assumes connection to BioRoot+Database.

---


### fetchLabGroupName

```
public java.lang.String fetchLabGroupName(int id)
```

:   Fetches an labGroupName given an id, assumes connection to BioRoot+Database.

---


### fetchGeneNames

```
public java.lang.String[] fetchGeneNames(int labGroupId)
```

:   Fetches geneNames given a labGroupId, assumes connection to BioRoot+Database.

---


### fetchMarkerNames

```
public java.lang.String[] fetchMarkerNames()
```

:   Fetches markerNames given an labGroupId, assumes connection to BioRoot+Database.

---


### getGeneId

```
public int getGeneId(java.lang.String geneName,
                     int labGroupId)
```

:   Fetches an geneId given a geneName and labGroupId (they aren't unique except within a labGroup),
    assumes connection to BioRoot+Database.

---


### getCellTypeId

```
public int getCellTypeId(java.lang.String name,
                         int labGroupId)
```

:   Fetches an tissueId given a tissue name and labGroupId (they aren't unique except within a labGroup)

---


### updateCommonAccessFields

```
public boolean updateCommonAccessFields(ReagentBean reagentBean,
                                        java.lang.String tableName)
```

:   Just updates fields that are accessible by all lab group memebers.

---


### updateCommonAccessFields

```
public boolean updateCommonAccessFields(ReagentBean bean,
                                        UserBean userBean,
                                        javax.servlet.http.HttpServletRequest request)
```

:   Just update the commonly accessible fields: lastUser, comments

---


### getCell

```
public java.lang.String getCell(java.lang.String sql)
```

:   Returns the first cell from an sql statement.

---


### getLastInsertId

```
public int getLastInsertId(java.lang.String table)
```

:   Returns the last inserted row auto increment number.

---


### getResultString

```
public java.lang.String getResultString()
```

:   returns the first column values from a result, for testing

---


### fetchSingleColumn

```
public java.lang.String[] fetchSingleColumn(java.lang.String tableName,
                                            java.lang.String columnName)
```

:   Returns a String[] of the items in a particular column.

---


### fetchColumn

```
public java.lang.String[] fetchColumn(java.lang.String statement)
```

:   Returns a String[] of the items in a particular column.

---


### fetchSingleColumn

```
public int[] fetchSingleColumn(java.lang.String sql)
```

:   Returns a int[] of the items in the first column.
    Returns a null if no entries are present.

---


### fetchLabGroups

```
public java.lang.String[] fetchLabGroups(java.lang.String[] labGroups)
```

:   Returns a sorted list of lab groups.

---


### convertOrganismNameToId

```
public int convertOrganismNameToId(java.lang.String name)
```

:   Converts and Organism name to and OrganismId.

---


### convertOrganismIdToName

```
public java.lang.String convertOrganismIdToName(int id)
```

:   Converts and Organism id to and Organism name.

---


### fetchMultipleCells

```
public java.lang.String[] fetchMultipleCells(java.lang.String sql)
```

:   Returns a String[] of the results.
    Returns a new String[]{""} if no entries are present.

---


### fetchUserName

```
public java.lang.String fetchUserName(int id,
                                      boolean firstNameFirst)
```

:   Returns firstName lastName or lastName, firstName given a user id number

---


### closeConnection

```
public void closeConnection()
```

:   Closes the Connection and subsequently the Statement and ResultSet objects

---


### isConnectionClosed

```
public boolean isConnectionClosed()
```

:   checks if the connection is open

---


### checkIP

```
public boolean checkIP(java.lang.String IP)
```

:   Checks to see if a given IP has exceeded the number of allowable failed logins.
    Will create a new IP entry if it's not found. Will reset entry and return true
    if 12hrs has passed.

---


### fetchReagentNames

```
public java.lang.String fetchReagentNames(int labGroupId,
                                          java.lang.String reagentType)
```

:   Fetches the sorted reagent names associated with a labGroup.

---


### buildSqlStatement

```
public static java.lang.String buildSqlStatement(java.lang.String reagentType,
                                                 UserBean user,
                                                 DBUtil bioRoot,
                                                 boolean descending)
```

:   Creates a sql statement given a UserBean and a boolean as to whether to add a DESC to the order by where appropriate.
    This also sets the Preference.ordered boolean if the sql contains an ORDER BY otherwise will set false. reagentType =
    Oligo, Plasmid, Strain...

---


### updateLoginFailure

```
public void updateLoginFailure(java.lang.String IP)
```

:   Increments the numberFailures for an ip in the Login table if it exists or
    creates a new record.

---


### executeSQLAdvance

```
public java.sql.ResultSet executeSQLAdvance(java.lang.String sqlQuery)
```

:   Execute and advance the ResultSet. Good for retrieving a single table row.

---


### getResultSetString

```
public java.lang.String getResultSetString(java.lang.String columnName)
```

:   Use to avoid a null return when attempting to fetch a ResultSet.getString().

---


### getResultSetInt

```
public int getResultSetInt(java.lang.String columnName)
```

:   Use to avoid a null return when attempting to fetch a ResultSet.getInt(). Sorta stupid

---


### fetchReagentCounts

```
public int[] fetchReagentCounts(UserBean user,
                                java.lang.String tableName)
```

:   Fetches the number of reagents in the users labgroup, and the number owned by the user.

---


### fetchOrganismConcat

```
public java.lang.String[] fetchOrganismConcat(int id)
```

:   Fetches concatinate of name and notes from Organism

---


### fetchMarkerConcats

```
public java.lang.String[] fetchMarkerConcats(int id)
```

:   Fetches concatinate of name and notes from Marker

---


### fetchCellTypeConcats

```
public java.lang.String[] fetchCellTypeConcats(int id,
                                               UserBean user)
```

:   Fetches concatinate of name and notes from CellType

---


### fetchGeneConcat

```
public java.lang.String[] fetchGeneConcat(int id,
                                          UserBean user)
```

:   Fetches concatinate of name and notes from Gene

---


### fetchRecentReagentConcats

```
public java.lang.String[] fetchRecentReagentConcats(int userId,
                                                    java.lang.String reagentName)
```

:   Fetches concatinate of name and notes from Plasmids

---


### fetchResultConcat

```
public java.lang.String[] fetchResultConcat(java.lang.String sql,
                                            int numColumns,
                                            java.lang.String divider,
                                            int lengthCutOff)
```

:   Returns a concatinate of the results where each String in the String[] represents a results
    table row where each column entry is separated by the divider String. If a particular column
    has no value, nothing is added, no double dividers. Use the lengthCutOff to limit the size of
    the Strings; a ... is added if it is trunkated. Returns new String[]{""} if no
    entries are present.

---


### fetchConcatMakeIdLast

```
public java.lang.String[] fetchConcatMakeIdLast(java.lang.String sql,
                                                int numColumns,
                                                java.lang.String divider,
                                                int lengthCutOff)
```

:   Returns a concatinate of the results where each String in the String[] represents a results
    table row where each column entry is separated by the divider String. If a particular column
    has no value, nothing is added, no double dividers. Use the lengthCutOff to limit the size of
    the Strings; a ... is added if it is trunkated. Returns new String[]{""} if no
    entries are present. The first query is assumed to be the id value and is always added to the end,
    even if the string was truncated. Thus the sql should look like "SELECT id, bla, bla FROM Table.."
    The result will look like bla,bla,id or bla, bla...,id
    This is a way of moving the ID to the end of the result yet trunkating in needed.

---


### getRowsEffected

```
public int getRowsEffected()
```


---


|  |  |  |
| --- | --- | --- |
| **Overview** \| **Package** \| **Class** \| **Use** \| **Tree** \| **Deprecated** \| **Index** \| **Help** | |  |
| **PREV CLASS**   **NEXT CLASS** | **FRAMES**    **NO FRAMES**     **All Classes** |  |
| SUMMARY: NESTED \| FIELD \| CONSTR \| METHOD | DETAIL: FIELD \| CONSTR \| METHOD |  |


---
